# UNDERSTANDING THE USE OF FLEXIBLE WORK ARRANGEMENTS AMONG OLDER INFORMAL CAREGIVERS

**DOI:** 10.1093/geroni/igac059.1113

**Published:** 2022-12-20

**Authors:** Shanika Koreshi, Fiona Alpass

**Affiliations:** Massey University, Palmerston North, Manawatu-Wanganui, New Zealand; Massey University, Palmerston North, Manawatu-Wanganui, New Zealand

## Abstract

The increasing provision of informal caregiving and the extension of working lives will result in many older workers combining paid work and informal caregiving responsibilities. Specific flexible work arrangement policies have been enacted in many countries to support working caregivers. Flexibility in the workplace has been suggested to promote prolonged employment among older workers. This study primarily focuses on the question of whether use of flexible work arrangements differs between caregivers and non-caregivers and how potential differences can be explained. Participants were 296 carers and 1611 non-carers (aged 55–70 years) who completed wave 8 of the New Zealand Health, Work and Retirement survey. The use of flexible work arrangements was analyzed based on five categories; Flexibility in number of work hours, flexible schedule, flexible place, options for time off, and other options. Hierarchical regressions were used to investigate caregiving as an independent predictor of use of flexible work arrangements after controlling for demographic and work characteristics. Results indicate that the studied informal caregivers on average used more workplace flexible arrangements than non-caregivers, both in flexible work hours, flexible schedules, and time off. The caregiver status difference in use of the three significant categories of flexible work arrangements can be explained by differences in socio-demographic and work characteristics. This difference in use of FWAs among older caregivers and non-caregivers warrants attention in discussions about prolonged employment and reconciliation of care and paid work.

